# A Case of Severe Asymptomatic Aortic Coarctation in an Adult Female

**DOI:** 10.1155/2011/535463

**Published:** 2011-07-10

**Authors:** Spyros Tsikrikas, Charalampos Charalampous, Konstantinos P. Letsas

**Affiliations:** ^1^Division of Cardiac Electrophysiology, Second Department of Cardiology, Evangelismos General Hospital of Athens, 10676 Athens, Greece; ^2^Second Department of Cardiology, Evangelismos General Hospital of Athens, 10676 Athens, Greece

## Abstract

Coarctation of the aorta is typically a disease of childhood and early adulthood, and there is a reduced life expectancy in patients who have not undergone correction. Survival to older age is rare, due to severe cardiovascular complications. We describe the case of a woman first diagnosed with coarctation of aorta at an advanced age.

## 1. Introduction

Coarctation of the aorta accounts for 5%–10% of congenital heart disease and occurs more frequently in males. It is usually diagnosed during childhood by routine examination of blood pressure and femoral pulse palpation [1]. We describe the case of a woman first diagnosed with coarctation of aorta at an adult age.

## 2. Case Report

**Figure 1:**
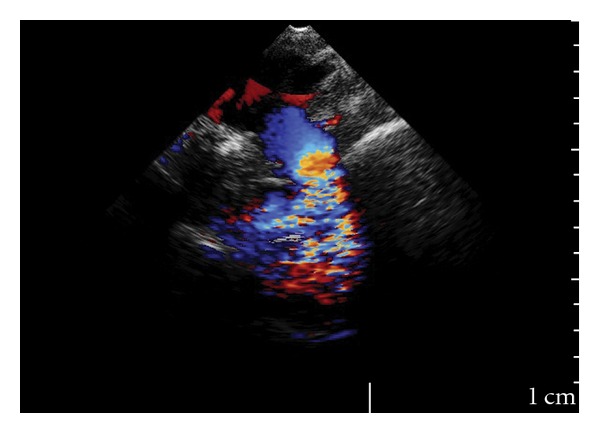
Transthoracic suprasternal view with a turbulent flow below the origin of subclavian artery.

**Figure 2:**
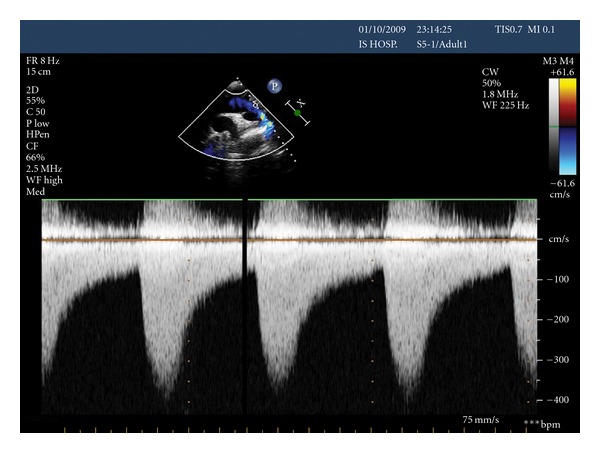
Doppler echocardiography demonstrating the coarctation gradient with a peak flow velocity in the descending aorta of 4.0 m/s, comparable to a gradient of 64 mmHg.

A 49-year-old female presented to Cardiology Department with chest discomfort, increasing fatigue, and exertional dyspnea. Her past medical history was significant for tobacco abuse and mild hypertension, and her brother was born with atrial septal defect, corrected at the age of 7 years old. On physical examination, she was identified a grade 2/6 systolic murmur in the apex and left second intercostals space that was radiated to the intrascapula area. The femoral pulses were palpable bilaterally; however, a radial-femoral pulse delay was noted. The blood pressure was 130/95 mmHg in the left arm and 100/75 mmHg in the right arm with a systolic pressure gradient of 25 mmHg between upper and lower extremities. Aortic coarctation was suspected, and a further investigation was performed. Results of routine blood chemistry and urine analysis were normal. Twelve-leads electrocardiogram revealed left ventricular hypertrophy, while the two dimensional (2D) echocardiography showed wall-motion abnormalities and the left ventricular ejection fraction was 0.35–0.40. The transthoracic echocardiography in suprasternal showed a turbulent flow (Figure 1) just below the origin of the subclavian artery, while the continuous wave Doppler revealed a peak flow velocity of 4,0 m/sec in descending aorta, (Figure 2) and the peak pressure gradient was estimated at 64 mmHg. The patient then underwent a transesophageal echocardiogram (TEE) that confirmed the findings and put the question of coarctation of aorta. The magnetic resonance angiography (MRA) showed severe coarctation of aorta below the origin of the left subclavian artery, together with poststenotic dilatation. MRA of the brain vessels did not detect any intracerebral aneurysm. The patient was then referred to cardiothoracic surgery to evaluate her candidacy for surgical or percutaneous therapy.

## 3. Discussion

Aortic coarctation is a congenital malformation that usually presents early in life and is often associated with congenital abnormal aortic valve. The mean survival for untreated patients is 35 years with a 25% survival rate beyond 50 years. The natural history of unrepaired coarctation of the aorta includes the development of systemic hypertension and subsequent morbidity and death from cardiovascular disease [2]. The age at correction is the most important factor for the relief of hypertension and long-term survival [3].

Despite the fact that the coarctation of aorta appears more often in young age and males, our case is referred to a mild-aged female. In addition, our patient was asymptomatic even though the aortic coarctation was severe, and though congenital heart disease runs out the family, aortic coarctation was first diagnosed at the fourth decade of her life. In literature, there are cases of aortic coarctation that was diagnosed over the age of 40 years [4–6]. In conclusion, clinical doctor must have in mind the possibility of appearance of a congenital heart disease at an advanced age.
